# Unique presentation of occult breast cancer with uterine cervix metastasis

**DOI:** 10.1002/ccr3.2306

**Published:** 2019-07-10

**Authors:** Emanuela Cimpeanu, Jibran Ahmed, Gabrielle Tricorico, Svetoslav Bardarov, Maxim Shulimovich, Nisha Lakhi

**Affiliations:** ^1^ Department of Internal Medicine Richmond University Medical Center Staten Island NY USA; ^2^ Department of Hematology and Medical Oncology Westchester Medical Center Valhalla NY USA; ^3^ Department of Obstetrics and Gynecology Richmond University Medical Center Staten Island NY USA; ^4^ Department of Pathology Richmond University Medical Center Staten Island NY USA; ^5^ Department of Hematology and Medical Oncology Richmond University Medical Center Staten Island NY USA; ^6^ Department of Obstetrics and Gynecology New York Medical College Valhalla NY USA

**Keywords:** asymptomatic cervical mass, breast‐conserving therapy, metastasis, occult breast cancer, systemic therapy, uterine cervix

## Abstract

We are reporting a case of occult breast cancer (OBC) diagnosed via biopsy of an asymptomatic cervical mass. While non‐OBC has occasionally been reported as metastatic to the uterine cervix, OBC never has, to our knowledge. Awareness of this presentation can be beneficial for a more expedite diagnosis and treatment.

## 1 INTRODUCTION

In women in the United States, breast cancer represents the most common malignancy and the second leading cause of cancer‐related deaths.^1^ OBC, however, is relatively rare.^2^ It is characterized by metastatic disease confirmed histologically as primary breast cancer, in the absence of a tumor mass on clinical examination and mammography.^3^ OBC often presents with nonspecific symptoms, owing to lack of disease in the breast, which can make diagnosis challenging.^4^ When discovered, it is usually metastatic to the axillary lymph nodes.^2^ Although less commonly reported, other metastatic sites include bone, liver, lymphatic system, skin, orbits, bone marrow, lung, and spleen.^5^, ^6^, ^7^, ^8^, ^9^, ^10^ To date, we have not found reports of OBC metastatic to the uterine cervix. A diagnosis of metastatic breast cancer, either occult or nonoccult, can be considered in certain patients presenting with an asymptomatic cervical mass.

## 2 CASE REPORT

A 76‐year‐old female presented with a sole complaint of 2‐month history of right hip pain. Her past medical history included essential hypertension, cardiovascular accident 19 years prior, type 2 diabetes mellitus, asthma, gastritis, and a cardiac arrhythmia requiring a pacemaker. Surgical history was significant for three cesarean sections. She had no family history of malignancy. Her menarche was at age 12 and menopause occurred at age 52. The patient reported a negative Papanicolaou test within the past year. Recent upper and lower gastrointestinal endoscopies were inconsistent with malignancy. Two months prior, the patient had bilateral breast ultrasound and mammogram, both of which did not identify any abnormalities. She denied abdominal discomfort and vaginal discharge. There was no palpable axillary, supraclavicular, or inguinal lymphadenopathy. Vaginal examination revealed a bulky cervix, an enlarged 16 weeks size mobile uterus and normal adnexa. Vital signs were as follows: blood pressure 130/70 mm Hg, heart rate 64 beats per minute, respiratory rate 14 breaths per minute, and temperature 98.5 F. Laboratory workup revealed white blood count 6.1 k/µL, hemoglobin 12.1 g/dL, blood urea nitrogen 25 mg/dL, creatinine 0.52 mg/dL, serum potassium 4.5 mmol/L, and serum calcium 9.3 mg/dL.

**Figure 1:**
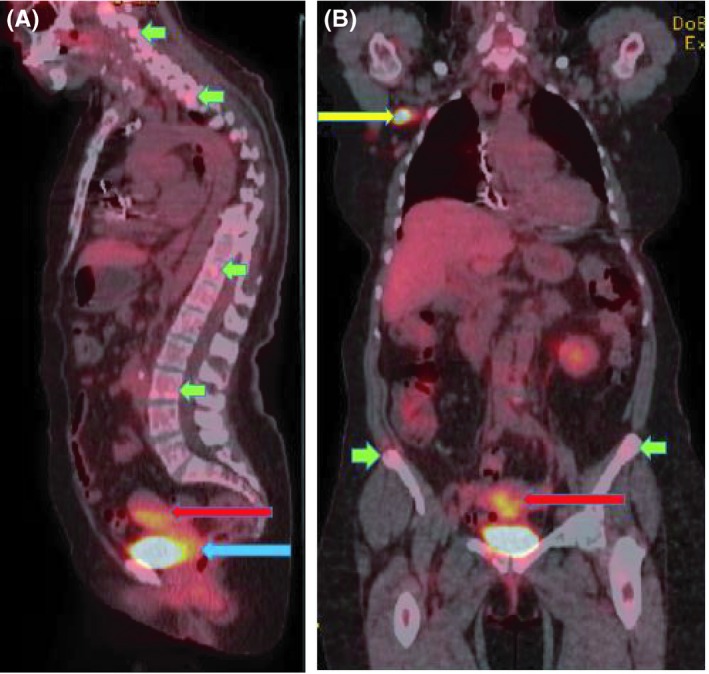
A, Coronal maximum intensity projection (MIP) image of a PET‐CT scan shows hypermetabolic activity in the cervix (blue arrow) and uterus (red arrow), with a maximum SUV of 4.9 (A). B, A right axillary lymph node measuring 2.6 × 1.5 cm (yellow arrow), with a maximum SUV of 12.7 is seen (B). Also present are multiple focal areas of uptake throughout the skeletal system (green arrows), predominantly at the level of the spine and bilateral iliac fossa, consistent with skeletal metastatic disease (A, B)

Further evaluation with abdominal and pelvic computed tomography (CT) showed diffuse metastatic disease with lytic bone lesions, an enlarged uterus containing a vague central hypodensity, and a 1.8 cm hypodensity with punctate calcification in the left adnexa. The patient was referred for positron emission tomography‐computed tomography (PET‐CT) (Figure 1A and B), which was significant for hypermetabolic activity in the cervix and uterus, skeletal metastatic disease, and right axillary adenopathy. There was no activity in the breast.

**Figure 2:**
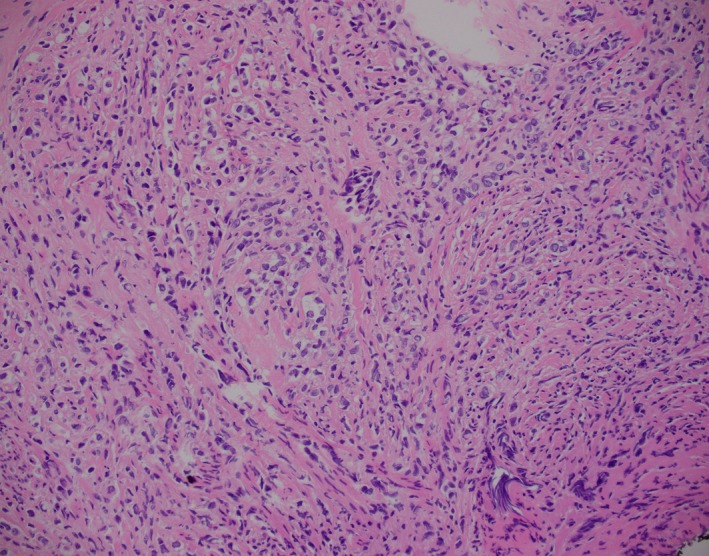
Invasive lobular breast carcinoma of cervix (hematoxylin and eosin staining; magnification, ×200)

Examination under anesthesia revealed a bulky cervical mass with parametrial involvement and extending into a fixed uterus measuring 16 weeks in size. Cervical biopsy (Figure 2) showed a cellular stroma infiltrated by a monotonous population of plasmacytoid cells arranged in single file, with increased nuclear‐cytoplasmic ratios and minimal nuclear pleomorphism. Small prominent nucleoli were observed, with no chromatin condensation and no mitotic figures. There was no necrosis. The malignant cells tested positive for CK‐7 and GATA‐3, and 91%‐100% positive for estrogen receptor (ER) and progesterone receptor (PR), supporting their mammary origin. Human epidermal growth factor receptor 2 (HER‐2) was negative. CT‐guided biopsy of the right axillary lymph node showed infiltration of tumor cells, morphologically identical to the ones observed in the cervix, with Ki‐67 of 10%‐15%.

The patient was started on palbociclib, letrozole, and zoledronic acid and, at 6 months follow‐up, has been clinically stable on this regimen. Clinical improvement was seen, with patient reporting resolution of pain. The patient will be monitored with surveillance imaging and further clinical examinations.

## 3 DISCUSSION

Occult breast cancers are rare entities. Only 0.4% of the 116 218 subjects listed in the Surveillance, Epidemiology and End Results database for the 2004‐2014 timeframe were diagnosed with OBC.^2^ Most patients were 50 years or older in age and presented with advanced stage cancers that were more likely than non‐OBC to be ER‐ and PR‐negative.^2^

To our knowledge, this is the first reported case of OBC metastatic to the uterine cervix. The uterine cervix is, in fact, very rarely involved even in metastatic non‐OBC, having been reported in anywhere from 0.8% to 1.7% of patients.^11^ When spreading to the genital tract, breast cancer preferentially affects younger women, likely secondary to their higher levels of estrogen,^12^ and it usually involves the ovary or endometrium.^13^ The lower predisposition for metastatic seeding in the uterine cervix likely has to do with its lower blood supply as well as the presence of an afferent lymphatic drainage system only.^14^ If the uterine cervix is involved, the usual presentation is vaginal bleeding, though the cancer can frequently be asymptomatic,^13^ such as in the case presented here. It is therefore important to take into consideration the fact that an asymptomatic cervical mass could in fact be a metastatic lesion.

As OBC is rarely encountered, there is a lack of evidence‐based medicine for its management, and no clear guidelines have been formulated. In OBC, when involvement is limited to the axillary nodes, mastectomy and breast‐conserving therapy (consisting of axillary nodal dissection plus radiotherapy) have proven equally effective in improving survival outcomes.^15^ While the 10‐year OS for either of these interventions is about 65%, patients who underwent axillary lymph node dissection only had a lower OS (58.5%).^15^ Interestingly, mastectomy did not prove superior to breast conservation therapy in improving cause‐specific survival. 15 Unfavorable outcomes, however, were more likely to occur with tumors that were ER‐positive and when 10 or more lymph nodes were involved. 15 Yet, after adjusting for confounders, OBC patients have been shown to have better overall survival (OS) than non‐OBC patients (*P* < .001).^2^

Little research has been available thus far on the treatment of widely metastatic OBC. Similarly to non‐OBC, systemic therapy, which can include chemotherapy, radiation therapy, and hormone therapy, is usually employed.^13^, ^16^ Factors that ought to be considered in choosing therapy include number and sites of metastatic lesions as well as performance status.^16^ In light of our patient's advanced breast cancer, she was started on palbociclib and letrozole. This regimen has proven efficacious for the treatment of ER‐positive, HER2‐negative breast cancer, such as our patient's, with a median progression‐free survival of 24.8 months vs 14.5 months (95% CI, 12.9‐17.1) in the placebo‐letrozole group.^17^ Benefits of palbociclib and letrozole occurred in patients of all ages, performance status, site of disease, previous therapy, or subtype of breast cancer.^17^ Even though adding palbociclib can lead to increased rates of myelotoxicity, supportive care, and decrease in dosages have proven effective measures for reducing such adverse effects.^17^ Thus far, 6 months after therapy initiation, our patient had been tolerating this regimen well, with no significant side effects.

In women with metastatic breast cancer but normal mammogram, such as our patient, magnetic resonance imaging (MRI) is a highly sensitive investigation (89% sensitivity)^18^ for identifying a primary breast lesion in anywhere from 62% to 70% of patients.^19^, ^20^ Due to health insurance limitations, our patient did not undergo an MRI. However, the standard for diagnosing OBC is the absence of a mass on clinical examination and mammography, both of which were negative.^3^ Moreover, the patient's PET‐CT (sensitivity 63%, specificity 91%)^18^ did not detect any breast lesions.

## 4 CONCLUSION

Metastatic breast cancer can be considered in the differential diagnosis of an asymptomatic cervical mass. A presentation of OBC with uterine cervical metastasis has not yet been reported in English literature. Further studies are needed to assess treatment options in OBC, as management is currently extrapolated from non‐OBC clinical trials.

## CONFLICT OF INTEREST

The authors declare that they have no competing interests.

## AUTHOR CONTRIBUTIONS

EC and JA: conceived and designed the case report and wrote the manuscript. GT: participated in writing the manuscript. SB: provided the pathology data as well as the histology image and its description. MS and NL: provided medical care for the patient, conceived and designed the case report and edited the manuscript. All authors have read and approved the final draft of the manuscript.
